# Long-Term Prophylaxis and Pharmacokinetic Evaluation of Intramuscular Nano- and Microparticle Decoquinate in Mice Infected with* P. berghei* Sporozoites

**DOI:** 10.1155/2017/7508291

**Published:** 2017-04-13

**Authors:** Qigui Li, Lisa Xie, Diana Caridha, Qiang Zeng, Jing Zhang, Norma Roncal, Ping Zhang, Chau Vuong, Brittney Potter, Jason Sousa, Sean Marcsisin, Lisa Read, Mark Hickman

**Affiliations:** Military Malaria Research Program, Walter Reed Army Institute of Research, Experimental Therapeutics Branch, Silver Spring, MD, USA

## Abstract

Decoquinate nanoparticle and microparticle suspended in an oily vehicle to retard drug release are evaluated for long-term malaria prophylaxis. Pharmacokinetic studies in normal animals and antimalarial efficacy in liver stage malaria mice were conducted at various single intramuscular-decoquinate doses for 2, 4, 6, or 8 weeks prior to infection with* P. berghei* sporozoites. The liver stage efficacy evaluation was monitored by using an in vivo imaging system. Full causal prophylaxis was shown in mice with a single intramuscular dose at 120 mg/kg of nanoparticle decoquinate (0.43 *μ*m) for 2-3 weeks and with microparticle decoquinate (8.31 *μ*m) injected 8 weeks earlier than inoculation. The time above MIC of 1,375 hr observed with the microparticle formulation provided a 2.2-fold longer drug exposure than with the nanoparticle formulation (624 hr). The prophylactic effect of the microparticle formulation observed in mice was shown to be 3-4 times longer than the nanoparticle decoquinate formulation.

## 1. Introduction

Decoquinate (DQ) is a 4-hydroxy quinoline compound that has been used as an anticoccidial drug in livestock such as cattle, sheep, and chicken, for many years without any signs of adverse effects. Besides its anticoccidial activity, it also has strong antimalarial activity against the blood and liver stages of* Plasmodium* sp. [1]. In addition, DQ kills developing gametocytes, the parasite stage responsible for malaria transmission. The mechanism of action of decoquinate is through selective and specific inhibition of the plasmodial mitochondrial* bc* (1) complex [2]. Decoquinate is structurally distinct to atovaquone, which is broadly used for malaria prophylaxis and treatment, and decoquinate demonstrates limited cross-resistance to atovaquone-resistant parasites [3]. Oral administration of a single dose of DQ has been shown to be effective as a prophylactic antimalarial drug, which reinforces the hypothesis that DQ is worthy of further development as an antimalarial in man [4]. Despite the demonstrated efficacy of DQ against malaria, it has never been clinically developed for use in man likely due to the very poor solubility and permeability of this compound.

Prophylaxis against malaria is essential to malaria control and elimination. Injection of a long-acting intramuscular depot of an antimalarial drug in an oil-vehicle may provide a better means of improving prophylaxis adherence, which, in turn, will decrease new malaria infections and reduce the rates of hospitalization due to recrudescent infections. In a recent publication, we discussed our work using nanonized and micronized decoquinate dispersed in oily carriers for causal prophylaxis. Our new nano- and microformulations injected intramuscularly show enhanced efficacy as causal prophylactics providing even better protection against malaria.

Previous efforts to develop soluble form of decoquinate revolved around making a DQ nanoparticle agent (0.24 *μ*m) which was manufactured through solid dispersion of DQ in polyvinylpyrrolidone [1]. The bioavailability of this DQ nanoformulation was increased 15-fold compared to standard DQ. In vivo imaging studies of mice infected with* Plasmodium berghei* sporozoites showed liver stage infections were inhibited by a dose of nanoparticle DQ as low as 1.25 mg/kg. By contrast, a dose of 40 mg/kg of standard DQ formulated as microparticles was needed to achieve similar results [1, 4]. While this DQ nanoformulation was demonstrably more soluble in water, showed increased bioavailability, and provided better causal efficacy, the elimination half-life of this DQ nanoformulation (23.93 hr, which is 3-fold longer than that plain DQ) is simply not a long enough period of prophylactic protection [4].

Formulations of long-term drugs are created to provide extended efficacy through continuous release of drug over time from a single injection of drug in a depot. Intramuscular or subcutaneous depot injections are generally formulations of drug in solid or oil-based carriers. Extended release drug formulations have a number of advantages over traditionally formulated drugs including reduced frequency of drug administration, better drug adherence, greater convenience, decreased adverse effects, more even drug dosing, and lower costs associated with healthcare. Drug is released from solid or oily drug depots very consistently over an extended period of time. A number of modifications to drug depots such as barrier coatings and variations in drug particle size can be used to control the dissolution of drugs in the depot [5].

Given the past results authors chose to advance this project by creating a number of slow-release oral and intramuscular DQ nano- and microparticle formulations [1, 4]. Those DQ formulations were created using a variety of carriers in aqueous polymeric dispersions to create release matrices capable of sustained drug dosing over time. We chose to use FDA-approved peanut oil as the vehicle for these formulations which were compared in vivo for both pharmacokinetic and pharmacodynamic effects. A microparticle suspension of DQ was also prepared as a comparator for both pharmacokinetic and pharmacodynamic studies.

## 2. Materials and Methods

### 2.1. Study Drugs, Sporozoites, Inoculation, and Viability Check

Decoquinate (DQ), methanol, n-butyl chloride, ethanol, and peanut oil were purchased from Sigma (Saint Louis, Missouri).

Luciferase-expressing* P. berghei* ANKA sporozoites were obtained from laboratory-reared female* Anopheles stephensi* mosquitoes from the Department of Mosquito Biology, WRAIR. The rearing of the mosquitoes and the preparation of* P. berghei* sporozoites from these mosquitoes were described previously by the method of Li et al. [6]. Sporozoites isolated from the same batch of mosquitoes were inoculated into C3H mice intravenously in the tail vein on the same day with 10,000 sporozoites suspended in 0.1 mL volume (Day 0). Sporozoites were counted with a hemocytometer and viability of sporozoites was assessed with fluorescein diacetate (50 mg/mL in acetone) and ethidium bromide (Sigma Chemical Co, St. Louis, MO, 20 *μ*g/mL in PBS). The viability of sporozoites thus prepared ranged from 89 to 97%.

### 2.2. Animals

Female 6-week-old C3H and ICR/CD-1 mice were obtained from Charles River Laboratories (Charles River Lab, MA). On arrival, the animals were acclimated in quarantine for 7 days. Mice were thus at 7 weeks of age at the initiation of these experiments. The animals were housed singly in a cage maintained in a room with a temperature range of 18–26°C, 34–68% relative humidity, and a 12-hour light/dark cycles. Standard rodent maintenance food was provided ad libitum during quarantine and throughout the study. All animal research was conducted in compliance with federal statutes, Army regulations, and the Animal Welfare Act principles stated in the Guide for the Care and Use of Laboratory Animals, NRC Publication, 2011 edition.

### 2.3. Preparation of IM Depot DQ in Nano- and Microsuspension

Nanoparticle suspension of DQ was prepared by particle size reduction using high-pressure homogenization and sonication using an oily vehicle, peanut oil. The dispersed DQ was suspended in oil and sonicated for 25–30 minutes (Elma S 40H, Singen, Germany) until the particle size was reduced to <15 *μ*m. The suspension was then homogenized using high-pressure homogenizer (Nano DeBEE, BEE international, Inc., South Easton, MA) at a pressure of 1500–2500 bars and a reflux temperature of approximately 30°C. Continuous cold-water flow was utilized for sample cooling. Particle size measurements were taken periodically throughout this process and high-pressure homogenization was continued until particle size reduction goals were achieved. Homogenization took place over a 3-4-hour period with a piston pump stroke frequency of 30-minute duration, followed by a pause of 10 minutes. The entire process entailed a total of 80–120 passes or homogenization cycles. The microsuspension DQ in oil was prepared by using probe sonication (Tekmar TM50, Cincinnati, OH) at approximately 40 W amplitude for 5 min in an ice water bath.

### 2.4. Matrices, Particle Size, and Drug Concentration

The particle size of the oily suspension was measured using a Horiba LA-960 particle size distribution analyzer (Kyoto, Japan), based on laser scattering. The calibration for peanut oil as a reference was taken before each measurement. All nano- and micro-DQ preparations were measured at a range of transmittance between 80 and 90%. Decoquinate formulation particle size was monitored throughout the preparation procedure and also before and after animal studies.

Drug concentrations and chromatography profiles of the drug molecule identity were examined using an Agilent HPLC (Agilent Technologies, Foster City, CA) with an isocratic mobile phase of 20% water and 80% acetonitrile (0.1% formic acid contained in water and acetonitrile, resp.). All drug preparations in aqueous system were diluted in methanol, vortexed, and then analyzed by HPLC in acetonitrile to ensure maximum compound extraction.

### 2.5. MCD_100_ Determination of IM-DQ during Long-Term Prophylactic Effects

**Figure 1:**
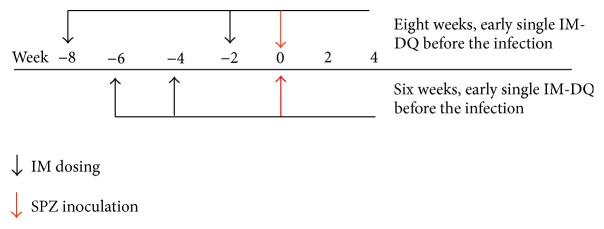
Diagrammatic sketch of the dosage regimens of the decoquinate (DQ) following single intramuscular injection in 2, 4, 6, and 8 weeks prior to an inoculation with 10,000* P. berghei* sporozoites intravenously in female C3H mice (*n* = 5).

For in vivo imaging studies, single intramuscular injections of DQ in nano- or microsuspension formulations were conducted 2, 4, 6, and 8 weeks prior to sporozoite inoculation (Figure 1). At 24, 48, and 72 hours after sporozoite infection, in vivo imaging studies of the livers of all infected mice treated with DQ or control preparations were conducted with an IVIS Spectrum (Perkin Elmer, Hanover, MD) to assess the burden of liver parasitemia. To assess blood stage parasitemia, parasite counts were conducted by flow cytometry. 4-Methyl primaquine, which is of 3-fold prophylactic potency than primaquine, was used as a positive control and a vehicle control group was also included as a negative control. All dosing solutions were prepared based on the body weight of mice. Both DQ nano- or microsuspensions were administered as single intramuscular injections administered with a 26-gauge needle to deep leg muscles. DQ nano- and microformulation concentrations were delivered in a volume of 100–200 *μ*L. ranging from 80 to 240 mg/kg of DQ by body weight. Prophylactic efficacy of* P. berghei* infected C3H mice was assessed by challenging these mice with 10,000 luciferase-expressing* P. berghei* sporozoites per mouse that had previously been treated with a single intramuscular DQ formulation injection administered 2, 4, 6, and 8 weeks earlier

The minimum curative dose (MCD_100_) in 100% of animals dosed was defined as the lowest dose which cured all animals in a group at any time during the first 30 days of the follow-up period.

### 2.6. In Vivo Imaging System (IVIS) Studies

IVIS studies of rodents infected with luciferase-expressing* P. berghei* mice were conducted, as described previously, using a Perkin Elmer IVIS Spectrum. Bioluminescence assessments were conducted on all animals at 24, 48, and 72 hours after sporozoite injection. To conduct in vivo imaging studies, mice were injected intraperitoneally with 150 *μ*L of luciferin at a concentration of 150 mg/kg body weight (Gold Biotechnology, St. Louis, MO). Three minutes after luciferin IP injection, the mice were anesthetized using inhaled isoflurane and positioned ventral side upon a heated platform inside the IVIS instrument. The mice were given isoflurane through nose cones throughout the IVIS imaging study. The IVIS camera exposure times utilized were 1 and 5 minutes for the 24-, 48-, and 72-hour time points with a large binning setting and an *f*-stop = 1. The IVIS Living Image software (version 4.0) was used to quantitate the bioluminescence in photons per second observed from the whole animal or the liver region. 3D bioluminescent imaging tomography was performed using sequential images taken with filters ranging from 580 to 660 nm [6].

### 2.7. Flow Cytometry (FCM)

Starting at 6 days after infection, all mice were assessed for blood stage parasitemia which was quantitated using flow cytometry conducted with an FC500 MPL flow cytometer (Beckman Coulter, Fullerton, CA). The green photomultiplier tube and filter setting were used for these studies (520–555 nm filter settings for the green PMT and greater than 580 nm settings for the red PMT). Infected erythrocytes, uninfected erythrocytes, and leukocytes were gated on logarithmic forward/side dot plots

The method of FCM sample preparation has been previously described previously [7, 8]. In brief, a small 3 *μ*L sample of blood was obtained from the tails of all mice. The blood was transferred into 0.3 mL of 1% heparinized isotonic buffer (PBS saline). 1 mL of 0.04% of glutaraldehyde was added to fix each sample, and samples were incubated for 60 minutes at 4°C followed by centrifugation at 450*g* for 5 minutes. The supernatant was removed by aspiration, and the cells were resuspended in 0.5 mL PBS buffer supplemented with 0.25% (v/v) Triton X-100 for 10-minute incubation at room temperature. After centrifugation, the permeabilized cells were resuspended in 0.5 mL of RNAse at 1 mg/mL concentrations and incubated for at least 2 hours at 37°C to ensure complete digestion of reticulocytes which are at high concentrations due to anemia associated with P*. berghei* infection. 20 *μ*L of YOYO-1 dye at a concentration of 2500 ng/mL was added to the 0.5 mL sample volume to create a final dye concentration of 100 ng/mL of YOYO-1.

### 2.8. Pharmacokinetic (PK) Studies

PK studies were performed after single injections of DQ formulations administered intramuscularly. Three male ICR mice per time point were acquired, aged 7 weeks, and were dosed with IM depot DQ nano- and microsuspensions at 120 mg/kg. The formulated drugs were injected at a volume of 100 *μ*L in one site, and a maximum of two injections were conducted with higher doses (240 mg/kg). At each time point, plasma and liver samples were collected. Whole blood was collected by cardiac puncture. Blood samples were collected in lithium heparin tubes within 0 h (baseline) prior to drugs administration and at 0.5, 1, 2, 4, 8, 24, 72, 144, 192, 240, 312, 360, 408, 480, 552, 720, and 888 h after drug administration. Plasma was obtained from the whole blood by centrifugation, and all samples were immediately preserved on dry ice and stored at −80°C until they were analyzed.

### 2.9. LC-MS/MS Analysis

Sample preparation was conducted by adding twice the normal volume of acetonitrile containing indomethacin as an internal standard (IS). The samples were mixed for 1 minute and centrifugation for 5 minutes, and the supernatant was transferred to an autosampler injection vial prior to separation by LC/MS/MS. Chromatography was performed using a Surveyor pump (Thermo Scientific, Waltham, MA) coupled to a Waters XTerra MS C18 with 50 mm × 2.1 mm id, 3.5 *μ*m particle size columns (Waters Corp., Milford, MA). The mobile chromatography phase consisted of a water/0.1% formic acid (Solvent A)/acetonitrile/0.1% formic acid (Solvent B) gradient. The gradient was set to begin at 2% B, rising to 98% B from 1 min to 3.5 min, held steady for 2 min, then returned immediately to its starting composition, and allowed to equilibrate for 1.5 min. Flow rate of samples through the column was set to 300 *μ*L/min. Samples were injected using an HTC PAL autosampler (LEAP Technologies, Carrboro, NC). Tandem mass spectrometry was performed using a TSQ Quantum AM (Thermo Scientific).

Standard curve and quality control (QC) samples were generated by adding known amounts of DQ and IS to mouse plasma samples, and all samples were injected at a volume of 40 *μ*L. Plasma standard curves were prepared via serial dilutions starting from 500 ng/mL. Serial dilutions of QC samples included samples at low and high DQ concentrations (10 and 100 ng/mL).

### 2.10. PK Parameter Determination

Drug concentrations of DQ versus time curves were prepared for both plasma and liver samples for each mouse. PK parameters for DQ in plasma were calculated using a compartmental analysis performed with Phoenix WinNonLin/Phoenix (version 6.4; Certara Corp., Mountain View, CA). Maximum plasma concentration (*C*_max_) and time to maximum concentration (*t*_max_) of DQ were obtained from the plasma drug concentration-time curves. The elimination half-life (*t*_1/2_) was determined from ln⁡2/*k*_el_, which is the elimination rate constant. The area under the curve (AUC) and the area under the first moment curve (AUMC) were estimated by the linear trapezoidal rule with extrapolation to infinity based on the concentration of the last time point divided by the terminal rate constant. Mean clearance rate adjusted by bioavailability (CL/*F*) was calculated by dividing the dose by AUC_inf_ for intravenous injection for plasma samples. Mean residence time (MRT) was calculated by dividing the area under the first moment curve (AUMC) by AUC. The volume of the central compartment adjusted by bioavailability (*Vz*/*F*) was determined as the product of CL/F and MRT.

### 2.11. Data Analysis and MIC Determination

The minimum inhibitory concentration (MIC) is the lowest concentration in the plasma of an antimalarial compound that will completely inhibit the visible growth of parasites resulting in a malaria cure. Plasma concentrations in the plasma derived from PK data of animals deemed cured were used to help define the MIC [9].

In the in vivo imaging experiments, causal prophylaxis activity, sporontocidal activity, parasite clearance, causal cure, delays in patency, and time to recrudescence were calculated as described previously [10, 11]. The data were generally found to fit a normal distribution. Means and standard deviations of photon measurement were calculated. Coefficients of variation were calculated as a percentage by dividing the standard deviation by the mean value.

## 3. Results

IM-DQ in nano- and microsuspensions was prepared by the procedure involving oily dispersion of the drug with particle size reduction by sonication or high-pressure homogenizer. To ensure that the formulation process with sonication and high-pressure homogenizer had not been destroyed or changed the DQ molecule, chromatography profiles such as mass quantity and retention time of drug compounds were analyzed by HPLC after completion of all preparations. The results showed that none of the preparations showed a changed in the DQ chromatography profile, suggesting the chemical structure of DQ was unaltered.

### 3.1. The Particle Size and Stability of IM-DQ Formulations

For all preparations, the particle size was measured using a Horiba LA-960 particle size distribution analyzer. For IM-DQ microsuspension preparations, 30-minute sessions of probe sonication were applied to minimize particle size to 5.34–11.31 *μ*m. The final batch of microsuspension IM-DQ was found to have a particle size of 8.31 *μ*m after combining different lots into one. This direct probe sonication was more effective for IM-DQ in oil than using a homogenizer with an open slotted generator, and this method yielded a mean particle size of 45.23 *μ*m. The nanoparticle sized (0.28–0.67 *μ*m) IM-DQ formulations were prepared with a combination of methods to include oily dispersion and sonication for 30 minutes followed by high-pressure homogenization at a pressure between 1500 and 2500 bars for 90 homogenization cycles. The final batch of the IM-DQ nanosuspension created through combination of different lots was measured to have a particle size of 0.43 *μ*m. In order to see the change in particle size of various preparations over time, an accelerated stability study of prepared nano- and microsuspensions was carried out at a temperature of 4°C for a period of up to 3 months. Accurately weighed amounts of samples were placed into glass vials with aluminum-lined caps and stored in a microprocessor-controlled humidity chamber, and the samples were then characterized as a function of exposed time. Generally, the particles in oily suspension dosed in animals were stable for at least 3 months.

### 3.2. Long-Term Prophylactic Efficacy of IM-DQ Nano- and Microsuspensions

Real time in vivo imaging to determine the timing and level of luminescence measured from luciferase expression of sporozoites development in the liver has previously been described [6, 10]. Our previous work has shown the C3H mouse model is very susceptible to liver infections with* P. berghei* sporozoites, and the extent of liver schizont growth can be readily assessed by measuring bioluminescent signals from the liver regions of intensity (ROI) at 24 and 48 hours and the whole body region at 72 hours after intravenous sporozoite inoculation. Five mice per group were treated with single IM-DQ in nano- or microparticle formulations administered as a single intramuscular injection at concentrations ranging from 80 to 240 mg/kg body weight. The mice were challenged intravenously with 10,000 luciferase-expressing* P. berghei* sporozoites administered to animals treated 2, 4, 6, and 8 weeks earlier with a single IM-DQ injection.

#### 3.2.1. IM-DQ Nanosuspensions

All mice treated with a single IM-DQ nanosuspension injection at a dose of 80, 120, or 240 mg/kg were shown to have different prophylactic outcomes during the treatment period of 2–8 weeks (Table 1). After IM-DQ nanoparticle injection at 80 mg/kg, one of five animals developed a blood stage infection during the 2-week period, suggesting that this formulation and a dose of 80 mg/kg have less than two weeks of prophylactic activity. When the single dose was increased to 120 mg/kg administered two weeks prior to sporozoite challenge, a low positive luminescence with 97.9% of suppression was observed at 24 hours in the liver stage period; afterward the photon signal was observed to be negative at 48 and 72 hours in all animals. In those mice, no animal developed a blood stage parasitemia during the 30-day period after challenge (Table 1). With the exception of the two-week group, complete cures of all animals were not observed with the 120 mg/kg dose administered 4, 6, or 8 weeks prior to sporozoite challenge. Accordingly, a dose of 120 mg/kg of nanoparticle IM-DQ should provide a minimal curative dose in all animals (MCD_100_) with a prophylactic effect of two-week duration. When the dose of IM-DQ nanosuspension was doubled to a single 240 mg/kg injection, the duration of causal prophylaxis was observed to be up to 8 weeks (Table 1).

#### 3.2.2. IM-DQ Microsuspensions

**Figure 2:**
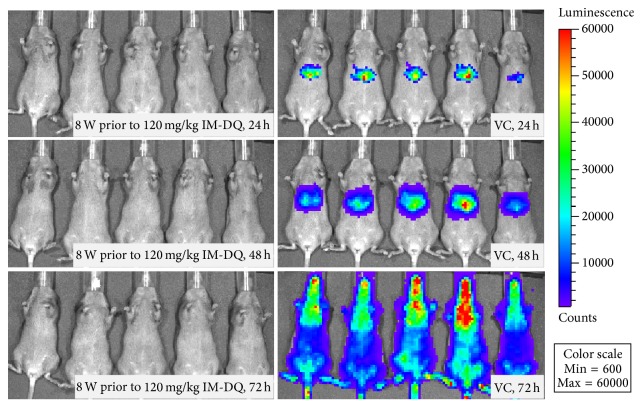
Representative in vivo bioluminescent images of C3H mice shown at different time points after injection of 10,000 sporozoites. Rainbow images show the relative levels of luminescence ranging from low (blue), to medium (green), to high (yellow/red). Luminescence levels (photons/sec) of livers in whole mice at 24-, 48- (liver stage), and 72-hour (blood stage) time points following single intramuscular dosing treated with IM-DQ in macrosuspension at a MCD_100_ dose of 120 mg/kg 8 weeks prior to malaria infection (8 W, left) and oily vehicle control (VC, right) after sporozoite infection intravenously at day 0. Normally,* P. berghei* sporozoites reside in the mouse liver for 44–52 hours after infection (*n* = 5).

Animals treated with a single IM-DQ microsuspension injection at a single dose of 120 mg/kg were shown to have full causal prophylaxis during treatment periods of 2–8 weeks prior to sporozoite challenge (Table 1). In the untreated animals, there was a strong growth in bioluminescence signal at 48–72 hours when compared to mice assessed at 24 hours (Figure 2, right). All mice treated with IM-DQ microsuspension injections at a dose of 120 mg/kg showed no liver stage luminescence during the observation period from 24 to 72 hours after infection. In addition, none of these animals developed blood stage infections during a 30-day period of monitoring after challenge, suggesting that a single injection of IM-DQ microsuspension at a dose of 120 mg/kg is the MCD_100_ which provided causal prophylaxis for a period of 8 weeks (Figures 1 and 2). All of the animals treated with IM-DQ nanosuspension showed a full causal effect of only two-week duration and incomplete cures with partial causal prophylaxis after IM-DQ nanosuspension injection 4 to 8 weeks prior to challenge, suggesting the IM-DQ nanosuspension formulation is a less potent product providing a shorter period of causal activity than the IM-DQ microsuspension formulation.

### 3.3. Plasma PK Profile of DQ in Animals Treated with a Single Injection of IM-DQ Nano- or Microsuspension

**Figure 3:**
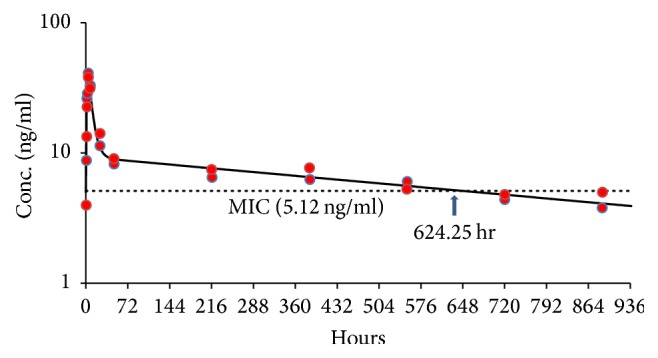
Mean plasma concentration-time profiles of intramuscular-decoquinate (IM-DQ) in nanosuspension measured by LC/MS/MS (red square) and computer fitted curves by pharmacokinetic parameters following single intramuscular injection at 120 mg/kg in ICR mice with minimum inhibitory concentration (MIC) of 5.12 ng/mL. The concentration* T* > MIC is 624.25 hr (*n* = 2).

The mean computer fitted plasma concentration-time curves following a single injection of IM-DQ nanosuspension in mice are shown in Figure 3. The PK parameter estimates of IM-DQ nanoformulations in plasma derived from animals are summarized in Table 2. In this study, injection of a single dose of IM-DQ nanoparticle suspension (0.43 *μ*m) at 120 mg/kg resulted in a mean half-life of 751.01 hr. The mice treated with a IM-DQ nanosuspension showed a low *C*_max_ of 36.58 ng/mL and a high AUC of 10,385 ng·h/mL following IM injection. In addition, administration of this formulation resulted in a large volume of distribution of 12,156 L/kg with a slow body clearance of 11.56 L/hr/kg, suggesting this DQ formulation resides in a large depot at the site of injection and corresponding slow elimination (Table 2, Figure 3).

**Figure 4:**
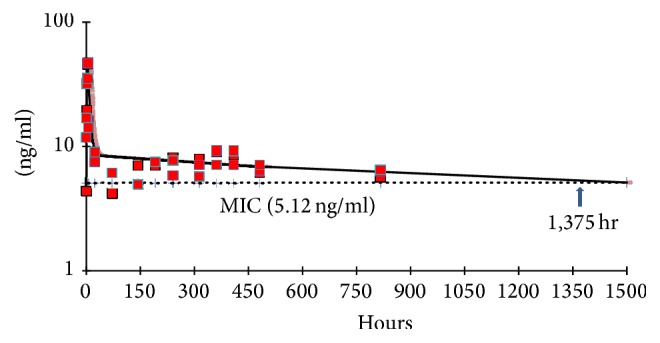
Mean plasma concentration-time profiles of intramuscular-decoquinate (IM-DQ) in microsuspension measured by LC/MS/MS (red square) and computer fitted curves by pharmacokinetic parameters following single intramuscular injection at 120 mg/kg in ICR mice with minimum inhibitory concentration (MIC) of 5.12 ng/mL. The concentration* T* > MIC is 1,375.00 hr (*n* = 3).

The mean computer fitted plasma concentration-time curves of DQ in animals after a single injection of IM-DQ microsuspension are shown in Figure 4. The PK parameter estimates after IM-DQ microsuspension injection in plasma are shown in Table 2. Injection of IM-DQ microparticle suspension (8.31 *μ*m size) at a dose of 120 mg/kg resulted in a very long DQ half-life of 1444.90 hr. The animals injected with the IM-DQ microsuspension had a high *C*_max_ of 45.35 ng/mL and a very high AUC of 18,311 ng·h/mL after IM injection. In addition, the microsuspension formulation provided a large body volume distribution of 13,403.78 L/kg with a slow body clearance of 6.57 L/hr/kg, suggesting the microsuspension formulation was residing in a large depot at the site of injection with slow elimination (Table 2, Figure 3).

The DQ microsuspension formulation was shown to have a much slower drug release at the same dose, 120 mg/kg, when compared to the DQ nanosuspension. The elimination half-life (1,444.90 hr) observed in animals treated with the IM-DQ microsuspension was 1.92-fold longer compared to the half-life (751.01 hr) observed in mice treated with the IM-DQ nanosuspension (Table 2), suggesting the large DQ particle size significantly prolonged the half-life decoquinate. The depot intramuscular DQ microsuspension formulation provides a slower drug release rate and a longer elimination time and also increases DQ drug concentration and bioavailability. The AUC of the IM-DQ microsuspension administered at a single dose of 120 mg/kg was shown to be 18,301 ng·h/mL, which is 1.76-fold higher than the AUC observed in animals injected with the IM-DQ nanosuspension. Body clearance data calculated from animals treated with the IM-DQ nanosuspension showed a CL/*F* of 11.56 L/hr/kg, which is almost twice as fast as the clearance observed in animals treated with the microparticle DQ formulation (6.57 L/hr/kg).

### 3.4. Liver PK Parameters in Animals Treated with a Single IM-DQ Nano- or Microsuspension

DQ has both blood and liver stage antiparasitic activity, and therefore the PK profile of IM-DQ in the liver tissue is important. The liver tissue distribution of IM-DQ in mice injected with the DQ nano- and microparticle formulations at a dose of 120 mg/kg was analyzed, and the PK parameter estimates are summarized in Table 3. The mean *C*_max_ and AUC_inf_ of nanoparticle DQ in the liver tissue were observed to be 114.63 ng/g and 127,674 ng·h/g, respectively, following a single IM administration. The ratio of the liver AUC to the plasma AUC in animals dosed with nanoparticle IM-DQ was calculated to be 12.29. The mean elimination half-life of nanoparticle DQ in the liver was observed to be 861.16 hours after a single IM injection. Following administration of a single dose of microparticle DQ formulation, the derived PK parameters in liver tissue showed a mean *C*_max_ and AUC_inf_ of 455.29 ng/g and 156,410 ng·h/g, respectively. The ratio of the liver AUC to the plasma AUC in animals dosed with IM-DQ was calculated to be 8.54. The mean elimination half-life of microparticle DQ in the liver was shown to be 1421.00 hours after IM injection.

*C*_max_ and AUC_inf_ of IM-DQ microsuspension in liver tissue were approximately 3.97-fold and 1.23-fold, respectively, higher than *C*_max_ and AUC_inf_ observed in animals injected with nanoparticle IM-DQ. The elimination half-life in animals treated with the IM-DQ in microsuspension was estimated to be 1.65-fold longer than the elimination half-life observed in animals treated with IM-DQ nanosuspension.

### 3.5. Determination of the MIC of IM-DQ Microsuspension

In vivo MICs play an important role in PK/PD evaluations to confirm a period of prophylaxis associated with treatment at a given antimalarial concentration and to monitor the activity of new antimalarial agents or formulations. The in vivo MIC is a critical determinant of the dose and duration of the activity of an antimalarial agent or formulation [9, 12]. Based on the efficacy studies conducted with the IM-DQ microsuspension, all mice treated with an IM-DQ microsuspension at a dose of 120 mg/kg in at a period of up to 8 weeks prior to* P. berghei* sporozoite challenge showed no liver stage luminescence during the observation period from 24 to 48 hours (liver stage) after infection. In addition, none of these animals developed blood stage infection during the period from 72 hours up to 30 days after challenge (Figure 2, left), suggesting that the lowest dose of IM-DQ microsuspension at a dose of 120 mg/kg is the MCD_100_. By combining the efficacy data over the 8-week (1,344 hours) period of effective causal prophylaxis against* P. berghei* with the PK concentration-time profile, the minimum inhibitory concentration (MIC) in plasma can be calculated. The relevant DQ concentrations in plasma at 1,344 hours were 5.08, 5.33, and 4.96 ng/mL in the animals treated with a single injection of IM-DQ microsuspension at a dose of 120 mg/kg. Accordingly, we calculate the minimum inhibitory concentration (MIC) with prophylactic effect at 5.12 ng/mL in the mouse plasma.

Assessments of PK/PD relationships for antimalarial effects have shown that, for some antimalarials, parasite killing is dependent on the duration for which the antimalarial drug exceeds the MIC (*T* > MIC). In this study, the period by which* T* > MIC is 624.25 hours (3.72 weeks) in animals injected with a single dose of IM-DQ nanosuspension (Table 2). In the mice treated with a single IM-DQ microformulation injection, the time above the MIC was shown to be 1375.00 hours (8.18 weeks, Table 2).

## 4. Discussion

Intramuscular depot injection is a special preparation of drug, which is slowly released into the body over a number of weeks, even months, following injection. It is important to know that the drug released into the bodies of subjects by a depot injection is exactly the same as the drug administered. Assuming the two are identical, the benefits and the side effects of a depot injection should be the same as they would be if you took the drug by mouth. In order to improve the slow-release profile of DQ and to extend its half-life for an extended period required for malaria prophylaxis, IM injection formulations were prepared to enhance the slow release of DQ, a poor water soluble compound, in an oily based vehicle. This study clearly shows that controlled release of a DQ suspension is possible using oil as a carrier, and the release control of DQ can be controlled through manipulation of the particle size. The selection of appropriate particle sizes was critical to achieving the maximum controlled-release condition desired.

The elimination half-life of 1,445 hr observed in mice treated with the IM-DQ microsuspension was 1.92-fold longer than the half-life of 751 hr observed in mice treated with an IM-DQ nanosuspension. In addition, the IM-DQ microsuspension demonstrated an elimination half-life 176-fold longer compared to the elimination half-life of 8.23 hours observed in mice treated with oral DQ suspended in water [1], suggesting that the slow-release formulation of IM-DQ in microsuspension significantly prolongs half-life of DQ. The advantage of oily depot intramuscular formulations not only increases the drug exposure time, but also increases drug concentration and bioavailability. The AUC of the IM-DQ microsuspension after a single dose of 120 mg/kg was shown to be 18,311 ng·h/mL, which is 1.76-fold higher than the AUC of nanosuspension IM-DQ in. After calculating the equal dose level, the AUC of IM-DQ is 230-fold greater than the AUC observed after administration of oral DQ in water [1].

The high concentration of DQ distributed in the liver is very important due to the obligate nature of liver schizont growth which begins the complex life cycle of* Plasmodium* parasites. In experimental animals [13, 14], a female* Anopheles* mosquito infected with* P. berghei* parasites feeds on a mouse and injects the parasites in the form of sporozoites into the bloodstream. The sporozoites travel to the liver and invade liver cells. Over 2 days (47–52 hrs, Figure 2), the sporozoites grow, divide, and produce tens of thousands of haploid forms, called merozoites, per liver cell. This multiplication can result in millions of parasite-infected cells in the host bloodstream, leading to illness and death. The analysis of antimalarial drug efficacy against liver stage malaria is therefore much more complex compared to assessment of drug efficacy against blood stage parasites. In the animals treated with the IM-DQ nanosuspension, the liver concentration (AUC) of DQ was 127,674 ng·hr/g, which is 12-fold higher than in the plasma AUC (10,385 ng·hr/mL). Similarly, in the mice injected with IM-DQ microformulation, the AUC observed in the liver (AUC = 156,410 ng·hr/g) was shown to be 8.5-fold larger than the plasma AUC (18,311 ng·hr/mL). Therefore, IM-DQ produced a much higher concentration of drug in the liver cells that contributes to the extensive period of prophylaxis observed against rodent malaria.

The IM-DQ microparticle suspension in peanut oil provided the longest period of prophylaxis over a period of 8 weeks which is far longer than the 2-3 weeks of prophylaxis observed after injection of the nanoparticle DQ suspension. The difference in the duration of prophylaxis may be due to the slower and longer release from the microparticle DQ formulation compared to the nanoparticle DQ formulation at the site of injection [15]. Due to longer DQ release and higher drug concentrations, the PK studies with both IM-DQ particles clearly show why the DQ microparticle suspension provides a longer period of causal prophylaxis than the nanoparticle suspension. Similarly, many studies have demonstrated that larger drug particles in an intramuscular formulation provide significantly higher drug exposure levels and longer drug exposure times [15–17]. Those results suggest that the DQ particle size in an oily suspension should act as a release controller which provides a means to design certain duration of prophylactic activity when administered to animals or, ultimately, humans [15]. The enhanced release of DQ administered as a long-release IM formulation provides a clear advantage over orally administered DQ which may facilitate long-term dosing for prophylaxis or radical cure of malaria as a consequence of the enhanced efficacy of this formulation and the enhanced compliance associated with an injection versus an orally administered drug.

In order to cure liver and blood stage malaria, antimalarial concentrations in blood (free plasma concentrations) must exceed the MIC for the infecting parasites until the last parasite is killed [12, 18]. Thus, the* T* > MIC is an important PK determinant of therapeutic outcome as the rate of parasite killing is determined by the concentration-effect relationship above the MIC for the infecting parasites and by the antimalarial concentration profile in the treated subjects.

In this study, we demonstrated the in vivo MIC associated with the two IM-DQ formulations tested, which is the critical determinant for achieving effective causal prophylaxis, directly relates to the overall dose required and directly influences the duration of prophylaxis achieved. In the animals treated with the IM-DQ nanosuspension, the* T* > MIC, 5.12 ng/mL, lasted 3.72 weeks, suggesting that full causal prophylaxis could be achieved for at least 3 weeks. However, we have proof of full causal prophylaxis of nanoparticle IM-DQ in two-week treated mice, but not in four-week treatment animals. Based on the time above the MIC, although the results suggest that the prophylactic duration of the IM-DQ nanoparticle formulation does not prevent malaria when administered 4 weeks prior to challenge, it may well be suitable for providing 3 weeks of prophylaxis prior to challenge given the* T* > MIC in those animals is longer than 3 weeks.

In other animals treated with a single injection of microsuspension IM-DQ at a dose of 120 mg/kg, the* T* > MIC is 8.18 weeks, suggesting that the period of causal prophylaxis could last for 8 weeks. As expected, the MIC in the plasma during this period completely inhibited parasite replication. As drug levels fall below the MIC, malaria parasitemia will rise, and a recrudescent infection can be detected over time with reduced drug concentrations below the MIC [12]. Causal cures in treated animals require all malaria parasites in the blood to have been killed before the drug blood concentrations fall below the MIC. For both of the IM-DQ formulations tested, single IM injections provide sufficient drug to sustain prophylactic concentrations (i.e., MIC) for a period long enough to eliminate infecting parasites.

Defining the in vivo MIC using the concentration-effect relationship provides a means to predict how much and for how long drug is required for treatment. A generally efficacious regimen is one that maintains blood concentrations above the MIC in all subjects until complete elimination of even the most drug-resistant naturally occurring infections. The in vivo MIC is not simply a theoretical concept, and the MIC in this study played a role in determining the effective causal dose and also provided a means to predict the duration of causal prophylaxis. Variability in MIC is not clear, and as the MIC represents both drug and host effects, we do not know yet how the MIC is affected by the host immune responses even in nonimmune patients. Host contributions to parasite clearance in malaria impact the duration and severity of the initial infection and so are likely to be lowest early in acute infections in previously unexposed individuals. In rodents, it is possible for different mouse strains to have different MICs because the different inbred mouse strains have different genetic backgrounds [19, 20].

## 5. Conclusion

An IM-DQ microsuspension was made by choosing appropriate formulation components and an appropriate particle size to provide long-acting prophylaxis in mice. The IM-DQ microsuspension formulation was found to provide the slowest release from the site injection and a prophylactic effect against* P. berghei* sporozoite challenge for a period of 8 weeks. In addition, DQ particles in the range of 8.31 *μ*m were shown to have a substantial impact on the duration of causal prophylaxis when compared to nanosized particle suspensions of 0.43 *μ*m. The enhanced efficacy and slowed drug release of a DQ microformulation may provide a method of providing long-term prophylaxis against malaria.

## Figures and Tables

**Table 1:** Long-term efficacy of depot IM-decoquinate (DQ) formulation (nano- and microsuspension) dosed with single intramuscular injection at 80, 120, or 240 mg/kg before treatment given 2, 4, 6, or 8 weeks early and monitoring by using in vivo imaging system (IVIS) in C3H mice (*n* = 5).

Test agents	Dose (mg/kg)	Regimen	Liver suppression rate (%)			Blood infection by FCM	Number of C3H mice			Effects
			24 h	48 h	72 h		Challenged	Protected completely	Causal prophylaxis	
IM nano-DQ (0.43 *µ*m)	120	−8 weeks	71.8	70.5	65.7	5/5	5	0	0/5	Mild suppression
	120	−6 weeks	79.0	90.8	92.5	4/5	5	1	1/5	Partial causal prophylaxis
	120	−4 weeks	89.4	97.6	99.6	1/5	5	4	4/5	Partial causal prophylaxis
	120	−2 weeks	97.9	100	100	0/5	5	5	5/5	Full causal prophylaxis
	80	−2 weeks	100	91.7	100	1/5	5	4	4/5	Partial causal prophylaxis

IM nano-DQ(0.43 *µ*m)	240	−8 weeks	100	100	100	0/5	5	5	5/5	Full causal prophylaxis
	240	−6 weeks	100	98.9	100	0/5	5	5	5/5	Full causal prophylaxis

IM micro-DQ (8.31 *µ*m)	120	−8 weeks	100	100	100	0/5	5	5	5/5	Full causal prophylaxis
	120	−6 weeks	100	98.8	100	0/5	5	5	5/5	Full causal prophylaxis
	120	−4 weeks	100	99.3	100	0/5	5	5	5/5	Full causal prophylaxis
	120	−2 weeks	100	99.6	100	0/5	5	5	5/5	Full causal prophylaxis

IVIS study: decoquinate (DQ) injected 2, 4, 6, and 8 weeks early to the challenge with 10,000 sporozoites (SPZ) intravenously in C3H female mice, which will be monitored with IVIS on days 1, 2, and 3 after SPZ inoculation. The blood parasitemia will be measured by flow cytometry up to 30 days after inoculation.

**Table 2:** Main pharmacokinetic parameters of decoquinate in oily suspension with nano- and microparticle following single intramuscular injection at 120 mg/kg dose level in mice plasma (*n* = 2-3).

Main PK parameters following IM injection	Nano-DQ (0.43 *µ*m) plasma	CV%	Micro-DQ (8.31 *µ*m) plasma	CV%
*C*_max_ (ng/ml)	36.58 ± 3.56	9.74	45.35 ± 8.09	17.84
*T*_max_ (hr)	3.52 ± 0.13	3.82	3.22 ± 0.90	27.86
AUC_inf._ (ng·hr/ml)	10,385 ± 405	3.91	18,311 ± 926	5.06
*t*1/2_distribution_ (hr)	4.84 ± 0.71	14.61	3.24 ± 1.51	46.59
*t*1/2_elimination_ (hr)	751.01 ± 28.72	3.82	1,444.90 ± 118.56	8.21
*V*ss/*F* (L/kg)	12,155.69 ± 844.54	6.95	13,403.78 ± 447.67	3.34
CL/*F* (L/hr/kg)	11.56 ± 0.45	3.91	6.57 ± 0.32	4.84
Minimum inhibitory concentration (MIC) (ng/ml)^*∗*^	5.12 ± 0.18	3.51	5.12 ± 0.18	3.51
*T* > MIC (hr)	624.25 ± 60.74 (3.72 weeks)	9.73	1375.00 ± 50.57 (8.18 weeks)	3.68

^*∗*^Value was calculated based on full causal prophylaxis effect for 8 weeks. IM: intramuscular; DQ: decoquinate; CV: coefficient of variation.

**Table 3:** Main pharmacokinetic parameters of decoquinate in oily suspension with nano- and microparticle following single intramuscular injection at 120 mg/kg dose level in mice liver (*n* = 2-3).

Main PK parameters following IM injection	Nano-DQ (0.43 *µ*m) liver	CV%	Micro-DQ (8.31 *µ*m) liver	CV%
*C*_max_ (ng/g)	114.63 ± 7.19	6.27	455.29 ± 46.03	10.11
*T*_max_ (hr)	3.36 ± 0.13	3.86	2.85 ± 0.39	13.68
AUC_inf._ (ng·hr/g)	127,674 ± 2,475	1.94	156,410 ± 15,076	9.64
*t*1/2_distribution_ (hr)	3.14 ± 0.27	8.59	1.98 ± 0.51	25.76
*t*1/2_elimination_ (hr)	861.16 ± 22.40	2.60	1,421.00 ± 40.26	2.83

MIC at 1,344 h (ng/ml)^*∗*^			47.35 ± 5.17	10.93

^*∗*^Value was calculated based on full causal prophylaxis effect for 8 weeks. IM: intramuscular; DQ: decoquinate.

## Abbreviations

PK: Pharmacokinetics
DQ: Decoquinate
IVIS: In vivo imaging system
PK/PD: Pharmacokinetics/pharmacodynamics
IM-DQ: Intramuscular-decoquinate
MCD_100_: Minimum curative dose in 100% of animals
ROI: Region of intensity
FCM: Flow cytometry
PMTs: Photomultiplier tubes
IS: Internal standard
QC: Quality control
MIC: Minimum inhibitory concentration
*T* > MIC: The duration for which the antimalarial drug exceeds the MIC.

## References

[1] Wang H., Li Q., Reyes S. (2013). Formulation and particle size reduction improve bioavailability of poorly water-soluble compounds with antimalarial activity. *Malaria Research and Treatment*.

[2] Da Cruz F. P., Martin C., Buchholz K. (2012). Drug screen targeted at plasmodium liver stages identifies a potent multistage antimalarial drug. *Journal of Infectious Diseases*.

[3] Nam T.-G., McNamara C. W., Bopp S. (2011). A chemical genomic analysis of decoquinate, a Plasmodium falciparum cytochrome b inhibitor. *ACS Chemical Biology*.

[4] Wang H., Li Q., Reyes S. (2014). Nanoparticle formulations of decoquinate increase antimalarial efficacy against liver stage Plasmodium infections in mice. *Nanomedicine: Nanotechnology, Biology, and Medicine*.

[5] Anselmo A. C., Mitragotri S. (2014). An overview of clinical and commercial impact of drug delivery systems. *Journal of Controlled Release*.

[6] Li Q., O'Neil M., Xie L. (2014). Assessment of the prophylactic activity and pharmacokinetic profile of oral tafenoquine compared to primaquine for inhibition of liver stage malaria infections. *Malaria Journal*.

[7] Xie L., Li Q., Johnson J., Zhang J., Milhous W., Kyle D. (2007). Development and validation of flow cytometric measurement for parasitaemia using autofluorescence and YOYO-1 in rodent malaria. *Parasitology*.

[8] Li Q., Gerena L., Xie L., Zhang J., Kyle D., Milhous W. (2007). Development and validation of flow cytometric measurement for parasitemia in cultures of P. falciparum vitally stained with YOYO-1. *Cytometry A*.

[9] Andrews J. M. (2001). Determination of minimum inhibitory concentrations. *Journal of Antimicrobial Chemotherapy*.

[10] Ploemen I. H. J., Prudêncio M., Douradinha B. G. (2009). Visualisation and quantitative analysis of the rodent malaria liver stage by real time imaging. *PLoS ONE*.

[11] Thiberge S., Blazquez S., Baldacci P. (2007). In vivo imaging of malaria parasites in the murine liver. *Nature Protocols*.

[12] White N. J. (2013). Pharmacokinetic and pharmacodynamic considerations in antimalarial dose optimization. *Antimicrobial Agents and Chemotherapy*.

[13] Mota M. M., Pradel G., Vanderberg J. P. (2001). Migration of Plasmodium sporozoites through cells before infection. *Science*.

[14] Meis J. F. G. M., Verhave J. P., Jap P. H. K., Meuwissen J. H. E. T. (1985). Transformation of sporozoites of Plasmodium berghei into exoerythrocytic forms in the liver of its mammalian host. *Cell and Tissue Research*.

[15] Leng D., Chen H., Li G. (2014). Development and comparison of intramuscularly long-acting paliperidone palmitate nanosuspensions with different particle size. *International Journal of Pharmaceutics*.

[16] Simon A., De Almeida Borges V. R., Cabral L. M., De Sousa V. P. (2013). Development and validation of a discriminative dissolution test for betamethasone sodium phosphate and betamethasone dipropionate intramuscular injectable suspension. *AAPS PharmSciTech*.

[17] Salem H. F. (2010). Sustained-release progesterone nanosuspension following intramuscular injection in ovariectomized rats. *International Journal of Nanomedicine*.

[18] MacGowan A. (2011). Revisiting Beta-lactams—PK/PD improves dosing of old antibiotics. *Current Opinion in Pharmacology*.

[19] Fortin A., Stevenson M. M., Gros P. (2002). Complex genetic control of susceptibility to malaria in mice. *Genes and Immunity*.

[20] Hernandez-Valladares M., Rihet P., Iraqi F. A. (2014). Host susceptibility to malaria in human and mice: compatible approaches to identify potential resistant genes. *Physiological Genomics*.

